# Merits and limitations of latent profile approaches to teachers’ achievement goals: A multi-study analysis

**DOI:** 10.1371/journal.pone.0284608

**Published:** 2023-04-20

**Authors:** Martin Daumiller, Stefan Janke, Ruth Butler, Oliver Dickhäuser, Markus Dresel

**Affiliations:** 1 University of Augsburg, Augsburg, Germany; 2 University of Mannheim, Mannheim, Germany; 3 Hebrew University of Jerusalem, Jerusalem, Israel; Georgia Southern University, UNITED STATES

## Abstract

Research on teacher goals has primarily followed a variable-centered approach, although person-centered approaches have inspired achievement goal research in other domains. The multiple goal perspective posits that individuals pursue different combinations of goals—goal profiles—that might be differentially adaptive or maladaptive. We investigate how beneficial goal profiles may be for research on teacher motivation, using data from three study sets (total *N* = 3,681) from different countries (Israel, Germany) and institution types (schools, universities). We analyzed whether psychologically meaningful, coherent, and generalizable goal profiles could be identified and compared the explanatory power of profiles and individual goals as predictors of teachers’ self-efficacy and work-related distress. Results showed six psychologically meaningful and largely generalizable goal profiles. Compared to individual goals, profiles only explained little differences in self-efficacy and work-related distress. Given these findings, we critically evaluate achievement goal profiles as a means to study effects of teacher goals.

## 1. Introduction

Research into teacher motivation has shown that teachers’ achievement goal pursuit matters, for teachers themselves as well as for their teaching outcomes. Studies conducted in different countries and in different types of institutions ranging from primary schools to universities have consistently documented that different achievement goals are differentially associated with teachers’ coping strategies [e.g., 1], emotional experiences [e.g., 2, 3], well-being at work [e.g., 4–6], learning behaviors [e.g., 7], and instructional practices [e.g., 8–11]. Nevertheless, the by now substantial body of empirical research on this subject has primarily relied on a variable-centered approach, which assumes population-homogeneity and considers linear associations between discrete goals and one or more theoretically relevant outcomes. Besides this approach, a multiple goal perspective has emerged within research on achievement goals [see 12, 13] postulating that different subgroups of individuals can be identified that pursue different combinations of achievement goals, or goal profiles, that might in turn be particularly adaptive or maladaptive. This perspective has inspired a large body of person-centered research on elementary [e.g., 14], secondary [e.g., 15, 16], and higher education students [e.g., 17].

Yet when it comes to teachers, achievement goal profiles have rarely been investigated and little is known about the power of such an approach for describing their achievement motivations and explaining differences in their cognitions and experiences. Besides studying the main effects of achievement goals, considering achievement goal profiles holds the potential to provide complimentary information for improving our understanding of how teachers pursue different goals and how these different combinations of goals matter. To this end, investigating differences in teachers’ self-efficacy beliefs and work-related distress can be considered as particularly insightful, given that these constructs are central, important, and well-researched aspects of teachers’ work [18] and are often considered in profile-based investigations in students [13]. Furthermore, knowledge on teachers’ achievement goal profiles is also essential for practical implications, as information on different subgroups of (differentially motivated) teachers is essential for group-specific interventions and training.

Given the lack of research in the teaching domain, the merits of person-centered approaches besides variable-based approaches for modeling achievement goal configurations within teachers are unclear. Therefore, we address the applicability of such an approach to teacher motivation by investigating the suitability of goal profiles for describing teacher motivation in a psychologically meaningful way and for predicting differences in teachers’ cognitions and experiences compared to variable-centered approach.

### 1.1 Achievement goal profiles as an approach to achievement motivation

The cornerstone of our reflections on teacher motivation constitutes the achievement goal approach, which provides an influential framework for explaining what motivates individuals in achievement situations. It has been applied to a broad range of populations such as students [e.g., 19], athletes [e.g., 20], employees [e.g., 21], and teachers [e.g., 22]. Within this, achievement motivation is defined in terms of qualitatively distinct types of goals characterized by the different end-states that individuals pursue in achievement situations [23]. In research on teacher motivation, four distinct types of achievement goals are commonly distinguished [e.g., 11, 22, 24, 25]: mastery goals (i.e., striving to develop one’s professional competence). In the present study, we focus on learning (approach) goals (defined by intraindividual competence gains) as a central component of the superordinate mastery goal construct [that also comprises task standards and avoidance valence, see 26], performance approach goals (i.e., striving to show high ability and outperform others), performance avoidance goals (i.e., striving to avoid appearing incompetent and performing worse than others), and work avoidance goals (i.e., striving to get through the day with little effort).

Several goal theorists have further differentiated teachers’ achievement goals, for example by considering relational goals (i.e., striving to create positive relationships with students; [10], learning avoidance goals (i.e., striving to not do worse than before; [27]), and task goals (i.e., striving to master a task, [27]; see [28], for an overview model). In the present study, we focus on the original four goals (mastery, performance-approach, performance-avoidance, and work-avoidance) proposed by Butler [22] as this framework has guided most of the research on teachers’ achievement goals to date and thus has a strong empirical foundation [2]. Importantly, these goals also align best with those most frequently used in person-centered studies on students’ achievement goal profiles [13], enabling us to compare the identified goal profiles to those found in prior research.

Research on goal profiles among students has largely stemmed from debates as to whether certain goals, or alternatively goal combinations, lead to (or at least are associated with) more effective learning [13]. The position that certain goals are superior to others is commonly labeled as the “mastery-goal perspective”. Researchers adhering to this perspective state that purely pursuing mastery goals elicits the most beneficial outcome pattern [29, 30].

In contrast, advocates of the “multiple-goal perspective” share the notion that achievement goals are not mutually exclusive [see 31]. Moreover, they argue that—besides for example specialized effects—a certain combination of goals may lead to the most beneficial outcome pattern. This perspective has primarily emerged from the mixed pattern of results for performance approach goals [13], which correlate with outcomes deemed desirable such as high achievement, as well as with undesirable outcomes such as increased test anxiety [see also 32, for an overview]. Advocates of the multiple goal perspective often claim that performance approach goals are especially beneficial when adopted alongside mastery goals, such that the strong endorsement of both goals is equally or even more beneficial than the endorsement of strong mastery goals alone [12, 33]. While this specific assumption can be tested with interaction terms within the General Linear Model, the multiple goal approach also sparked a more general debate as to whether variable-centered approaches are appropriate in the first place, given the number and complexity of possible goal patterns with potentially differential consequences [13]. These considerations led to the growing popularity of person-centered investigations into students’ goal profiles.

### 1.2 Merits and limitations of person-centered analyses into achievement goals

While variable-centered analyses allow for investigations into associations between variables within certain populations, person-centered analyses such as Cluster Analyses or Latent Profile Analyses (LPAs) enable researchers to identify subgroups of individuals that differ from one another regarding the investigated variables [13]. In line with prior research on achievement goal profiles, we use the term *person-centered* to refer to research that identifies and compares subgroups of individuals based on mean differences where individuals with similar expressions in a bundle of variables are grouped together. Person-centered analyses into achievement goals allow for insight into potential goal configurations (i.e., *goal profiles*) that differentiate groups of individuals based on how the various goals combine within individuals, while also maintaining the power of subsequent analyses into correlates of these goal profiles. This can be illustrated by comparing variable- versus person-centered approaches for the four goals that are typically used to characterize teachers’ achievement goals. Using a variable-centered approach, it would be necessary to consider four main effects, six two-way interactions, four three-way interactions, and one four-way interaction to investigate the impact of every possible goal composition [e.g. see 34]. Analyses into so many complex interactions on top of main effects likely face problems regarding statistical power [35] and interpretability [36]. Furthermore, variable-centered approaches rely on the assumption of population-homogeneity and linear interactions for all strengths to which goals are pursued. In contrast, person-centered approaches relax the assumption of population-homogeneity and allow for the identification of goal profiles that are potentially suitable to characterize groups within a certain population, usually resulting in a much smaller number of profiles (compared to the number of possible interactions). Further, Wormington and Linnenbrink-Garcia [13] noted in their meta-analysis on goal profiles that, compared to variable-centered approaches, person-centered approaches may be more useful to model the effects of performance approach and performance avoidance goals [which are often very strongly associated in both students as well as teachers, see 2, 32, 37]. Finally, person-centered approaches afford comparisons between individuals characterized by a certain goal profile and individuals characterized by another goal profile, allowing for a better understanding of how goal compositions relate to adaptive patterns of cognitions and experiences. This advantage is particularly relevant for examining the contrasting predictions of the “mastery goal” and “multiple goals” perspectives as the latter assumes that the combination of strong mastery and performance goals will be beneficial mainly to the extent that they combine also with weak performance-avoidance goals.

Despite these potential benefits, an important premise of using person-centered analyses to investigate achievement goal pursuit is that similar motivational profiles can be found across different contexts. While different contexts, such as different types of educational institutions that differently value certain standards or different countries with varying educational systems and emphases can go along with differences in the motivations of teachers active in these contexts [38, 39], the general psychological mechanisms motivating teachers should apply equally [40, 41]. If this is not the case, findings from different samples cannot be sufficiently compared [see 13, 42]. Further, person-centered analyses into achievement goal pursuit can only provide substantial insights into differences in cognitions and experiences if goal profiles do indeed explain meaningful amounts of variance compared to the main effects of the single goals, as suggested by advocates of the multiple goal perspective [see also 43 for a similar argument]. This notion, however, is rarely reflected in the methodology of studies examining goal profiles [15–17], as the necessary investigations would require combining and comparing person-centered and variable-centered analyses [see 44, for a comparison of person and variable centered approaches to explain differences in students’ changes in conceptual understanding]. Besides the extent to which profiles are generalizable and theoretically reasonable, a strong case for the usefulness of the multiple goal perspective in explaining differences in educational outcomes requires that the profiles explain substantial portions of variance compared to the main effects of the individual goals.

In their meta-analytical review including a large set of studies of students’ goal profiles, Wormington and Linnenbrink-Garcia [13] found that almost half of around 15,000 students (49.1%) were not characterized by qualitatively different configurations of stronger and weaker goals. Rather, there were quantitative differences in the general levels of achievement motivation such that the levels of all goals were average, high, or low. Wormington and Linnenbrink-Garcia [13] interpreted this finding to indicate that these different general levels of achievement goal pursuit may be as important as the relative strength of the individual goals for describing students’ goal pursuit. Using other motivational frameworks such as Self-determination Theory, person-centered approaches often document similar findings about the prevalence of qualitative and quantitative differences in motivational profiles [e.g., 45]. Taking a critical stance, one might interpret a large proportion of profiles only differing by quantitative differences as challenging a person-centered approach, as it highlights the necessity to compare mean values, which can be achieved more directly with variable-centered approaches. Moreover, as the quantitative differences in motivational profiles align primarily with older approaches that often defined achievement motivation as a one-dimensional, quantitative motivational force, one might even argue that such findings do not align well with the fundamental assumptions of the achievement goal approach and Self-determination Theory on the quality of motivation mattering [i.e., “which kind” of motivation, and not "how strong" motivation is, see 12].

In sum, a person-centered approach could advance research into teachers’ achievement goals—if it helps to differentiate teachers alongside goal profiles that are meaningful (i.e., characterized by qualitative and theoretically reasonable differences rather than quantitative differences in achievement motivation), generalizable (e.g., found in different groups of teachers), and explain substantial variance in other variables when compared to the main effects of the individual achievement goals.

### 1.3 Potential goal profiles within teacher populations

Beyond the finding that about half of the students in the reviewed studies could be characterized by the general strength of their achievement goals, Wormington and Linnenbrink-Garcia [13] found two profiles to frequently (but not always) emerge that were characterized to a stronger degree by qualitative rather than only quantitative differences regarding goal pursuit. In particular, these goal profiles allow for deeper comparison of the mastery goal perspective versus the multiple goal perspective. The first qualitatively differentiated goal profile identified in 62.5% of all investigated studies was indicated by strong mastery goals. According to the mastery-goal perspective, individuals that are characterized by this goal profile should show the most beneficial learning behavior when compared to any other goal profile. The second goal profile identified in 50% of all investigated studies was indicated by strong mastery and strong performance approach goals. This goal profile is of particular interest for scholars expecting interactive goal patterns following the multiple-goal perspective, as comparisons between individuals with this goal profile to other groups (especially with individuals characterized by exclusively strong mastery goals) could indicate whether the combination of strong performance approach goals and strong mastery goals is particularly beneficial. Replicating these two goal profiles within teacher populations would be of great interest in order to test the applicability of the mastery- versus multiple-goal perspectives within the population of teachers, as it is not clear without specific research on this topic whether this finding holds and can be extended to this population [see also 46 on the general relevance of replication in educational research].

When writing this manuscript, Subsequently, another study has investigated goal profiles in teachers [for a preprint, see 47]. While not directly relatable to the presented findings as the profiles also included relational goals, the authors identified profiles characterized by (a) high levels of approach goals, (b) high levels of mastery and relational goals, (c) high levels of performance goals, and (d) low levels of all goals the only published study on goal profiles in the teaching profession that we were aware of was reported by Kunst et al. [48], who analyzed achievement goals of 984 vocational training instructors from the Netherlands. The authors found that 50.1% were classified into a “diffuse” profile with average levels of all goals, 10.7% into a high mastery and performance approach goal profile, and 12.3% into a low performance approach and performance avoidance goal profile. These profiles are conceptually similar to the previously described profiles reported by Wormington and Linnenbrink-Garcia [13]. In line with the multiple goal perspective, Kunst et al. [48] found that teachers who were classified into the high mastery and performance approach goal profiles reported more information acquisition and feedback asking than those in the other profiles. However, it is also worth noting that Kunst et al. [48] did not identify a high mastery profile, but only a profile with moderate mastery goals and weak performance goals. Nevertheless, this affirms the notion of investigating goal profiles also in teachers and expecting, in principle, similar patterns as in students [see 42, for qualitative differences in motivational profiles in teachers using a SDT framework].

### 1.4 Relevant correlates for research into teachers’ goal profiles

Beyond the existence of achievement goal profiles in students, Wormington and Linnenbrink-Garcia [13] investigated whether students who were characterized by certain goal profiles also differed in other motivational variables and their social and emotional well-being. In the reviewed articles, they found the strongest support for the view that a high-mastery goal profile was most beneficial with regard to these outcomes when compared to any other goal profile. While this provides some support for the mastery-goals perspective within students, a conceptual replication in samples of teachers would support the generalizability of findings and the usefulness of person-centered approaches on achievement-motivation. With that being said, studies on teachers’ achievement goals that have applied the variable-centered approach frequently showed positive associations between mastery goals and other aspects of teachers’ motivation such as self-efficacy beliefs [49, 50] and teachers’ well-being [6, 11].

In order to relate our research to the broader picture of research into teachers’ goals, we chose to investigate two variables that are central to teachers’ cognitions and experiences, that matter for their work, and that have often been investigated in prior (variable-centered) studies of teachers’ achievement goals: teachers’ self-efficacy (reflecting an expectancy-based aspect of motivation aside from the goals that are grounded in the value-component of motivation) and work-related distress (as proxy for impaired well-being).

*Teachers’ self-efficacy beliefs* describe teachers’ self-ascribed capabilities to influence student learning [51]. Entailing the core aspects of teacher agency, visible as effort and perseverance when fulfilling instructional goals, self-efficacy beliefs are considered one of the key personal beliefs influencing teachers’ professional behaviors and student learning [52]. Teachers’ self-efficacy is positively related to both mastery and performance approach goals, while being negatively associated with performance avoidance goals and especially with work avoidance goals [e.g., 2, 24, 49, 53]. As far as we are aware, no empirical studies to date have investigated whether there is any effect of the goal composition beyond the additive influence of both goals. Fundamentally, we want to investigate whether teachers who are characterized by strong mastery and performance approach goals (multiple goals) experience substantially higher self-efficacy than those who merely report strong mastery goals or strong performance approach goals.

Besides self-efficacy, teacher well-being is a critical construct for schools and society that is linked to the effective functioning of schools, school improvement and implementation of educational reforms, staff commitment, and teacher absenteeism [54]. We understand well-being as a multifaceted and broad construct, entailing satisfaction as well as positive and negative affect [54, 55]. An important component that threatens the effective work of teachers are experiences of *work-related distress* [56]. These have often been operationalized as burnout experiences defined by feelings of exhaustion at work, reduced personal accomplishment, and depersonalization [57]. Research has confirmed strong links between achievement goals and both burnout [6, 9] and other measures of work-related distress such as occupational strain [58]. Mastery goals were negatively associated with experiences of work-related distress, and performance avoidance goals and work avoidance goals (the latter to an even greater extent) were positively related to such experiences. The pattern of results was more inconclusive for performance approach goals, which showed either positive associations [58] or, more typically, null effects [e.g., 9]. This leaves room for speculation whether associations may vary depending on feelings of distress can differently emerge from a combination of different (performance) goals. For example, performance approach goals might be associated with distress if combined with strong performance avoidance or work avoidance goals, while no such association, or possibly even a negative association, may be found if instead accompanied by strong mastery goals. A person-centered approach could shed light on these complex relations.

## 2. Research questions

Our overarching objective was to investigate achievement goal profiles of teachers and their meaningfulness compared to the insights provided by variable-centered approaches. Our first research question was whether it is possible to consistently identify achievement goal profiles, which indicate qualitative rather than quantitative differences in achievement goal pursuit, between subgroups of teachers (particularly in terms of strong mastery goals but not strong performance goals, and strong mastery goals combined with strong performance goals). One serious problem of the person-centered approach is that the emergence of some goal profiles might strongly depend on the respective sample. For instance, Wormington and Linnenbrink-Garcia [13] pointed out that half of the goal profiles identified in their meta-analysis only emerged in less than 25 percent of the investigated student samples. This means that we have to be cautious in over-interpreting results from single samples of teachers. To overcome this problem, we included a set of samples from different countries (Israel, Germany) and different teaching contexts (primary/secondary versus higher education) to investigate the robustness of the observed goal profiles. Specifically, we examined whether the profile patterns varied depending on systematic differences between these teacher populations (country, institution type) or whether we would find generalizable profiles across different populations.

As a second research question, we investigated whether teachers with different goal profiles also differed in their self-efficacy beliefs and experiences of work-related distress. Building on this, our third research question was whether the person-centered latent profile approach can yield insights into associations of teachers’ achievement goal pursuit with self-efficacy beliefs and experiences of work-related distress that cannot easily be obtained using a variable-centered approach. Specifically, we investigated the explanatory power of goal profiles and set this in relation to the additive main effects of the individual achievement goals.

## 3. Method

### 3.1 Procedure and sample

To examine the research questions, we used existing datasets from two different countries and two different educational contexts. In total, we used six datasets from studies that were conceptually similar regarding their focus on teachers, their cross-sectional design, and the measures used. Two studies were conducted with primary and secondary school teachers in Israel (*n* = 950 and *n* = 408), two studies with university teachers in Germany (*n* = 933 and *n* = 832), and two studies with secondary school teachers in Germany (*n* = 224 and *n* = 334). Table 1 provides an overview of all included datasets, reference publications, and details about their sample. As the two data sets from each of the three different educational contexts are very similar (not only regarding the sampled population, but also the measures; see Table 1), we combined them to yield three larger datasets (Israel-school, German-university, German-school) that were used to conduct the LPAs. Unlike some of the single datasets, these three combined datasets met the rule of thumb criteria of *N* = 500 that is frequently considered as a minimum sample size necessary for sufficiently high accuracy in identifying the correct number of latent profiles [59, 60].

**Table 1:** Overview of the included datasets.

Studies used	*N*	Population	Achievement goal measure	Other measures
Israel-school study set				
Study 1 [10]	950	Israeli inservice primary and secondary school teachers	Butler [22] scale (“I would feel that I had a successful day in school if …”): Mastery (4 items, ω_h_ = .74, e.g., „I saw that I was developing as a teacher and teaching more effectively than in the past”); Performance approach (4 items, ω_h_ = .83, e.g., “my classes did better on an exam than those of other teachers”); Performance avoidance (4 items, ω_h_ = .64, e.g., “no one asked a question in class that I couldn’t answer”); Work avoidance (4 items, ω_h_ = .79, e.g., “some of my classes were cancelled”).	Self-efficacy (11 items, ω_h_ = .89, e.g., insert sample items here); Work-related distress (10 items, ω_h_ = .85, e.g., “I feel emotionally drained by my work”; [61]).
Study 2: [62]	408	Israeli inservice school teachers	Butler’s [22] scale (see above): Mastery (4 items, ω_h_ = .73); Performance approach (4 items, ω_h_ = .82); Performance avoidance (4 items, ω_h_ = .65); Work avoidance (4 items, ω_h_ = .74).	Work-related distress (4 items, ω_h_ = .93, e.g., “I feel emotionally drained by my work”; [61]).
German-university study set				
Study 3 [63]	933	German university teachers	Daumiller et al. [28] scale (“In my current teaching activities …”: Mastery (4 items, ω_h_ = .88, e.g., “my goal is to expand my professional and methodological knowledge as much as possible”); Performance approach^a^ (8 items, ω_h_ = .92, e.g., “my goal is to teach better than my colleagues”); Performance avoidance^a^ (8 items, ω_h_ = .93, e.g., “my goal is to not teach worse than my colleagues do”); Work avoidance (4 items, ω_h_ = .94, e.g., “it is my goal to have the least amount of work as possible”).	Self-efficacy^b^ (12 items, ω_h_ = .86, e.g., “How well can you respond to difficult questions from your students?”; adaption from Nie et al. [64]); Work-related distress^c^ (17 items, ω_h_ = .92, e.g., “I feel emotionally drained by my work”; MBI, [65]).
Study 4: [38]	832	German university teachers	Daumiller et al. [28] scale (see above): Mastery (4 items, ω_h_ = .92); Performance approach^a^ (8 items, ω_h_ = .91); Performance avoidance^a^ (8 items, ω_h_ = .94); Work avoidance (4 items, ω_h_ = .93).	Self-efficacy ^b^ (12 items, ω_h_ = .82, same scale as in Study 3); Work-related distress^c^ (22 items, ω_h_ = .85, same scale as in Study 3).
German-school study set				
Study 5: [24]	224	German inservice secondary school teachers	Nitsche et al. [24] scale (“In my vocation, I aspire to …): Mastery (18 items, ω_h_ = .92, e.g., “improve my pedagogical knowledge and competence.”); Performance approach (6 items, ω_h_ = .90, e.g., “show my colleagues that I deal better with critical lessons than other teachers”); Performance avoidance (6 items, ω_h_ = .91, e.g., “conceal from my colleagues when I do something less satisfying than other teachers”); Work avoidance (6 items, ω_h_ = .85, e.g., ”get through the day with little effort.”).	Self-efficacy (7 items, ω_h_ = .73, e.g., “When I try really hard, I am able to reach even the most difficult students”; [66]); Work-related distress (15 items, ω_h_ = .88, e.g., ”The pressure under which I am working is too big”; [67]).
Study 6: [68]	334	German inservice secondary school teachers	Nitsche et al. [24] scale (see above): Mastery (9 items, ω_h_ = .88); Performance approach (3 items, ω_h_ = .84); Performance avoidance (3 items, ω_h_ = .84); Work avoidance (3 items, ω_h_ = .80).	Self-efficacy^d^ (5 items, ω_h_ = .74, “Considering the challenges that my occupation poses to me, I get along well in my job”; [69]); Work-related distress^e^ (6 items, ω_h_ = .84, same scale as in Study 5).

*Note*.

^a^ This scale distinguishes between appearance and normative aspects of performance and assesses them separately; for the investigation at hand, we combined both aspects to correspond to the measures of the other studies.

^b^ This scale distinguishes self-efficacy for instruction, classroom management, and motivation; again, we combined all three aspects as a general measure of teaching self-efficacy.

^c^ This scale distinguishes emotional exhaustion, depersonalization in the workplace, and reduced personal accomplishment; we again combined all three aspects.

^d^ In this dataset, self-efficacy in a narrow sense was not measured, but rather core-efficacy beliefs in the form of self-concept. We conducted the analyses twice, once with and once without this measure to ensure that our findings were not affected by the use of this measure that is slightly different to the other self-efficacy scales, albeit strongly related to self-efficacy beliefs.

### 3.2 Measures

An overview of the measures in the different studies including sample items and internal consistencies can be found in Table 1. All McDonalds Omega values were > .60 which is typically deemed as a minimum value for evaluating acceptable internal consistency [see 70].

#### Achievement goals

Regarding teachers’ achievement goals, mastery approach goals (measured by learning approach goals as a core aspect of the superordinate mastery goal construct), performance approach goals, performance avoidance goals, and work avoidance goals were measured using three established scales for the three study sets, namely scales by Butler [22], Daumiller et al. [28], and Nitsche et al. [24]. These scales are conceptually similar, however differ slightly in their measures. Most notably, Daumiller et al. [28] distinguished performance goals further, as to whether they focus on appearance aspects or normative aspects. As the other two scales include items that address both of these aspects, we aggregated both appearance and normative goals for the studies that used the Daumiller et al. [28] measure to enhance comparability with the other two scales. In addition, the Nitsche et al. [24] scale distinguishes three facets of the content of mastery goals as well as four facets of different addresses of performance goals. These were also aggregated on the overall goal level [see 28, for a similar approach].

#### Self-efficacy beliefs

To assess teachers’ self-efficacy, the Israel-school studies used a Hebrew translation of Tschannen-Moran & Hoy’s [71] short measure of teacher self-efficacy, the German-university studies an adaption of the scale by Nie, Lau, and Liau [64] and the German-school studies a German version of the teacher self-efficacy scale by Schwarzer and Hallum [66]. To enhance comparability of all scales across the different study sets, we aggregated the subscales that distinguished between different aspects of self-efficacy beliefs on overall scale level.

#### Work-related distress

Regarding teachers’ work-related distress, the Israel-school studies used measures based on the Maslach Burnout Inventory for teachers [61, 65] that were developed and validated for teachers in Israel by Friedman and Farber [72]. The German-university studies also used a scale based on the MBI, and the German-school studies used the perceived overload subscale by Enzmann and Kleiber [67]. We aggregated the MBI measures that distinguished different components of work-related distress (emotional exhaustion, cynicism, reduced personal accomplishment) as a composite measure to ensure comparability of all scales between study sets.

### 3.3 Missing values

Overall, there was relatively little missing data (< 2.1% for each variable, for each dataset) that was handled using the full-information maximum likelihood estimation (FIML) and the EM-algorithm for all analyses [73].

### 3.4 Analyses

As the individual studies used different scales that did not always have the same number of answer options (see Table 1), we first *z*-standardized all variables (except for gender) for each individual dataset. For the LPAs, we followed the procedure outlined by Asparouhov and Muthén [74] using Mplus 8.1 [75]. Based on the teachers’ answers to their mastery, performance approach, performance avoidance, and work avoidance goals, we first specified two latent profiles. We subsequently increased the number of profiles until the increase in model fit no longer indicated specifying another profile and losing model parsimony. Based on the range of profiles found in prior research, we considered up to 9 latent profiles. Models were estimated using 5,000 random sets of start values, 100 iterations for each random start, and 200 best solutions retained for final stage optimization [76]. All solutions converged on well-replicated loglikelihood values. Changes in model fit were investigated with the Bayesian information criterion (BIC) and the sample-size-adjusted BIC (SSBIC). We also report the Akaike information criterion (AIC) for informational purposes but do not use it for model evaluation as it has a tendency for overextraction [see e.g., 77]. The exact fit values for a particular model were not relevant for deciding how many profiles to select. Instead, we analyzed the progressions of these model fit indices across the different number of profiles by visually inspecting the development of these fit indices analogously to the interpretation of scree plots [78] and selecting the profile after which the decrease in model fit was substantially lower compared to the previous profiles [79]. In addition, we used the Vuong-Lo-Mendell-Rubin likelihood ratio test (VLMR) as well as the Lo-Mendell-Rubin adjusted likelihood ratio test (LMR) to compare models with adjacent numbers of profiles. A significant *p*-value of these two tests indicates that a particular profile solution fits the data better than a model with one profile less. Finally, we also considered model parsimony (preferring simpler solutions over more complex ones), the statistical adequacy of the solution [e.g., absence of negative variance estimates; 80], and ease of interpretation (preferring solutions that yield profiles with qualitative differences and not solely mean levels, as well as profiles that can be sensibly interpreted). Subsequently, we used the posterior distributions to determine the most likely profile membership for each teacher (i.e., the profile to which an individual most likely belongs). The respective classification error of this procedure is reflected in the entropy values, with high values indicating small error.

We conducted this procedure for each for the three study sets (Israel-school, Germany-university, Germany-school). We then compared the number of profiles found and their configuration between these three study sets.

Next, we used the profile membership obtained from these analyses to examine the association of goal profiles with self-efficacy and work-related distress. To this end, we conducted equality tests of means across profiles using the BCH procedure [81]. Subsequently, we compared the exploratory power of goal profiles with the individual goals through structural equation modelling. Model 1 investigated goal profiles as predictors and Model 2 investigated the individual achievement goals. In each model, self-efficacy and work-related distress were regressed on these predictors. Achievement goals, self-efficacy, and work-related distress were estimated as latent variables based on item parcels as indicators [using the item-to-construct method, two parcels were used for each construct; see 82]. Again, we conducted these analyses separately on the level of the three study sets in order to allow for further insights into the generalizability of our findings.

We provide all code and data underlying our results in an open repository (https://osf.io/vrtsf/?view_only=e1eb3e81ab9b4c39a16530b354e74df5).

## 4. Results

Descriptive statistics for all analyzed datasets are presented in Table 2.

**Table 2:** Descriptive statistics for all included datasets (displayed are percentages for the proportion of female and means and standard deviations for the remaining characteristics).

	*%* Female	Years of teaching experience	Mastery (learning approach) goals	Performance approach goals	Performance avoidance goals	Work avoidance goals	Self-efficacy	Work-related distress
Israel school teachers								
Study 1: [10]	91	15.90 (9.51)	4.13 (0.62)	3.43 (0.91)	2.94 (0.77)	2.56 (0.88)	3.89 (0.56)	2.63 (0.63)
Study 2: [83]	88	14.04 (9.63)	4.17 (0.61)	2.07 (1.97)	2.73 (0.88)	2.18 (0.93)	–	2.65 (0.87)
German university teachers								
Study 3: [84]	49	–	4.23 (0.71)	3.36 (0.93)	3.69 (0.99)	1.70 (1.02)	3.67 (0.57)	1.82 (0.73)
Study 4: [85]	36	11.54 (9.13)	4.29 (0.76)	2.89 (0.99)	3.44 (1.15)	1.58 (1.01)	3.70 (0.51)	2.14 (0.50)
German school teachers								
Study 5: [24]	67	13.7 (11.8)	4.17 (0.51)	2.42 (0.91)	2.39 (0.95)	2.33 (0.87)	3.78 (0.52)	2.21 (0.61)
Study 6: [77]	72	11.13 (11.6)	4.34 (0.48)	1.99 (0.90)	2.28 (0.98)	2.34 (0.96)	4.08 (0.50)	2.85 (0.78)

*Note*. *N* = 3,681 teachers. For comparability of the descriptive statistics, goals, self-efficacy, and work-related distress have been rescaled to 1–5. “–” indicates that this variable was not assessed in the respective study.

### 4.1 Number of profiles

The results of the LPAs for the three study sets are presented in Table 3. We estimated and compared models comprising between two and nine profiles.

**Table 3:** Overview of LPA profile solutions.

Number of profiles	Fit indices			Likelihood ratio tests (*p* values)		Entropy	Number of teachers in profiles								
	BIC	SSBIC	AIC	VLMR	LMR		*1*	*2*	*3*	*4*	*5*	*6*	*7*	*8*	*9*
Israel-school study set															
2	14,831	14,790	14,763	< .001	< .001	.66	789	569							
3	14,721	14,664	14,627	< .001	< .001	.63	754	312	292						
4	14,705	14,632	14,585	.08	.08	.55	409	379	302	268					
5	14,684	14,595	14,538	.56	.56	.62	467	294	290	285	22				
6	14,692	14,587	14,519	.03	.04	.64	476	304	256	188	100	34			
7	14,699	14,578	14,501	.19	.19	.65	374	353	254	208	92	74	3		
8	14,713	14,576	14,489	.21	.21	.67	362	355	234	185	117	53	49	3	
9	14,726	14,574	14,476	.61	.61	.70	359	354	181	170	79	74	71	67	3
German-university study set															
2	17,880	17,839	17,809	< .001	< .001	.79	1,254	511							
3	17,486	17,429	17,387	.001	.002	.83	1,217	370	178						
4	17,200	17,127	17,074	.002	.002	.80	1,019	356	245	145					
5	17,020	16,931	16,867	.003	.003	.74	651	556	236	203	119				
6	16,899	16,795	16,719	.04	.04	.76	657	524	200	189	158	37			
7	16,871	16,750	16,663	.35	.36	.77	646	515	191	177	172	52	12		
8	16,820	16,683	16,584	.59	.59	.78	647	484	195	179	106	86	53	15	
9	16,782	16,629	16,519	.18	.19	.78	661	396	186	168	152	73	72	45	12
German-school study set															
2	6,095	6,053	6,038	< .001	< .001	.73	286	271							
3	6,058	6,001	5,980	.11	.12	.74	289	207	61						
4	6,027	5,954	5,928	.56	.57	.80	288	209	57	3					
5	6,026	5,937	5,905	.07	.07	.80	195	190	144	27	2				
6	6,030	5,925	5,887	.52	.53	.81	195	189	35	28	8	3			
7	6,034	5,914	5,870	.35	.36	.81	189	175	128	38	19	6	3		
8	6,041	5,904	5,855	.54	.54	.79	180	154	125	50	27	14	6	2	
9	6,052	5,900	5,844	.01	.01	.82	185	154	118	50	27	16	6	1	1

*Note*. BIC = Bayesian information criterion. SSA-BIC = sample-size-adjusted BIC. AIC = Akaike information criterion. VLMR = Vuong-Lo-Mendell-Rubin likelihood ratio test. LMR = Lo-Mendell-Rubin adjusted likelihood ratio test.

For the Israel-school study set, the differences in AIC, BIC, and SSBIC fit values were small after a three-profile solution and negligible after the six-profile model (see S1 Fig in S1 File for a visualization). For subsequent (i.e., seven and more profile solutions) models, we did not observe a good class discrimination, meaning that the new profiles were of low frequency and primarily characterized by differences in the general strength of goal pursuit but not so much by qualitative differences in the identified profiles. The three-profile solution was composed by three profiles that indicated whether mastery goals were endorsed alongside weak, moderate, or strong performance approach, performance avoidance, and work avoidance goals. The six-profile solution, however, indicated qualitatively more complex goal profiles that provided deeper insight into fluctuations within mastery goals as well. This is why we retained the six-profile solution, which provided an entropy of .64.

For the German-university study set, the fit differences pointed to a six-profile solution (see “knee” points for 2 and 6 profiles in S1 Fig in S1 File), as did the likelihood ratio tests. We did not observe a good class discrimination for more goal profiles, and therefore we retained the six-profile solution, which provided an entropy of .76.

For the German-school study set, the fit differences were small after a six-profile model, while the likelihood ratio suggested a two-profile model that primarily reflected mean level differences in performance goals (see S3 Fig in S1 File). The changes of the fit indices gradually became smaller after the two-profile solution, while the emerging profiles were still primarily characterized by mean level differences in performance goals (see S3 Fig in S1 File). Beginning with the six-profile solution, BIC values increased, while the changes in SSBIC and AIC values were small. In terms of content, the six-profile solution was the most meaningful; further profile solutions did not provide a good class discrimination. Although only a few teachers were classified into some of the profiles beyond a three-profile solution, we therefore again retained the six-profile solution to also allow for comparability with the other two study sets. The selected six-profile solution provided an entropy of .81.

In addition to these analyses, we also estimated the latent profiles on the level of an overall dataset that integrated the three datasets. The results from these additional findings are presented as supplementary materials (see S1 and S2 Tables, and S2 Fig in S1 File) and are to be interpreted with caution, as the three study sets contribute unequally to it with regard to sample size (with the German-university study sets being over- and the German-school study sets being underrepresented). Also on this level, both the decrease in fit indices and the likelihood ratio tests clearly spoke for the six-profile solution. This was further supported by low class discrimination in seven or more profile solutions and meaningful mean levels for the six-profile solution.

### 4.2 Configuration of profiles

**Fig 1:**
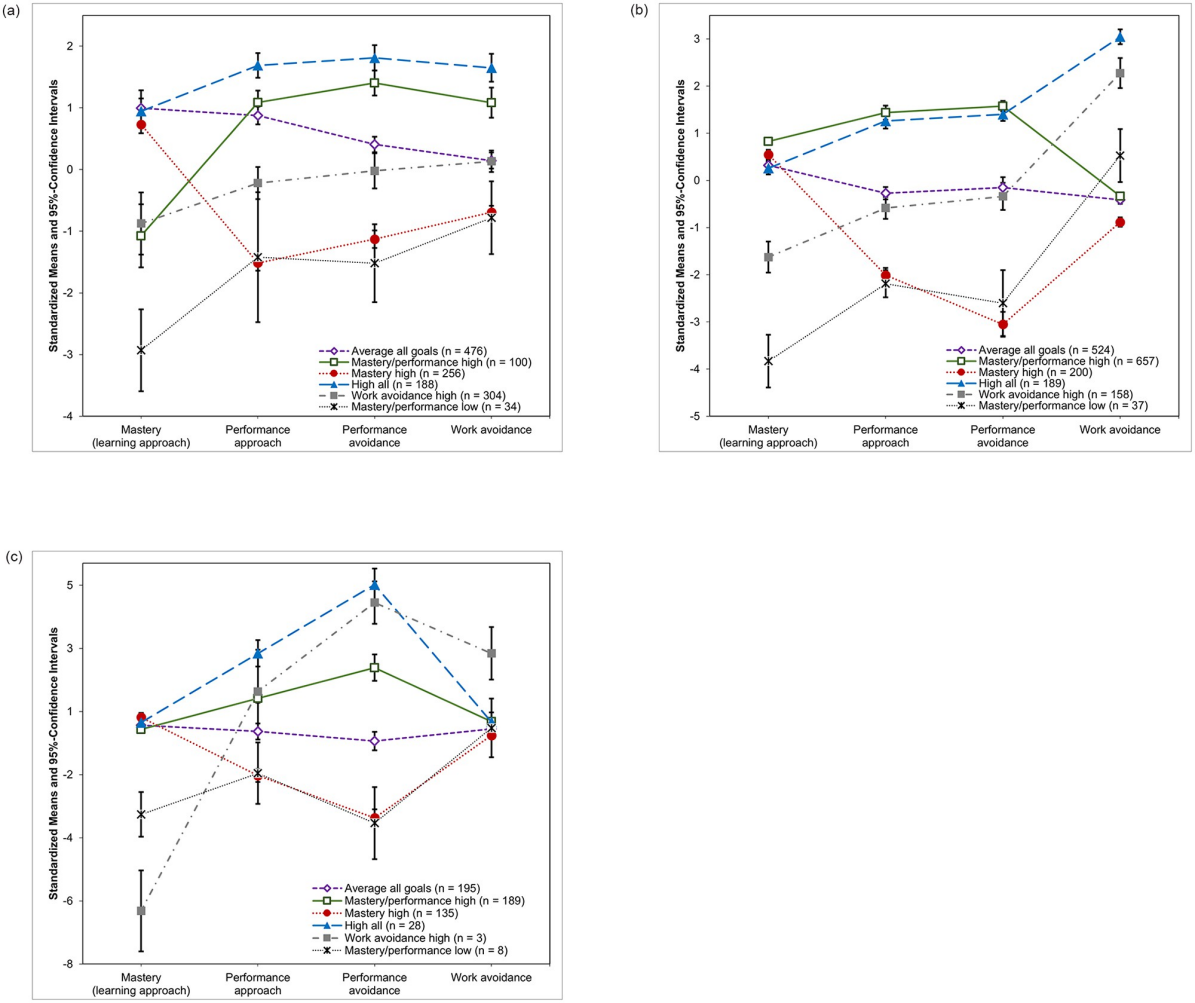
Goal means for the six latent profiles (with 95% confidence intervals). (a) Israel school teachers. (b) German university teachers. (c) German school teachers.

Fig 1 shows the means of the achievement goals for the six-profile solutions for the different study sets. The profiles were similar for the different study sets, particularly between the Israeli-school and the German-university study sets. We observed a group of teachers that pursued as strong mastery goals as most of the other teachers and average levels of the other three goals (**♢**). Overall, most teachers were classified into this profile. In the second profile, the teachers exhibited similar levels of mastery goals, but low levels of the other three goals (●). This profile was also similar across all three study sets and contained a substantial number of teachers. Another profile that was very similar across all three study sets entailed weak mastery and weak performance goals and average levels of work avoidance goals (**x**). Overall, only relatively few teachers were classified into this profile.

The configurations of the remaining three profiles varied a bit more across the study sets: First, there were two profiles with strong performance approach and performance avoidance goals. In the German-university study set, these had similarly strong levels of mastery goals but differed in the level of work avoidance goals, while in the Israel-school study set they differed in the level of mastery goals while work avoidance goals were similar between both profiles. In the German-school study set however, these two profiles also differed in the quantitative level of performance goals. Finally, the last profile was characterized by rather weak mastery goals and average to strong performance and work avoidance goals. This profile was more pronounced in the German-school study set than the other two.

On the overall dataset level, we found very similar profiles as in the three study sets, with average goal levels (**♢**; *average all goals*; overall: 39% of participants), strong mastery and weak performance and work avoidance goals (●; *mastery high*; 15%), weak mastery, weak performance, and average work avoidance goals (**x**; *mastery/performance low*; 2%), strong mastery, performance, and work avoidance goals (▲; *high all*; 13%), strong mastery and performance, but weak work avoidance goals (◻; *mastery/performance high*; 25%), as well as average mastery and performance goals combined with strong work avoidance goals (◼; *work avoidance high*; 6%).

It is worth pointing out that there were profiles that were characterized to the same extent by mastery (and work avoidance) goals, but had either strong, average, or weak performance goals. Indeed, these quantitative differences in performance goals characterized the three most prevalent profiles that around three fourths of the overall sample was classified into. Interestingly, we did not identify any profiles that were characterized by different levels of performance approach and performance avoidance goals. In somewhat similar vein, the high all and mastery/performance high profiles primarily differed by the extent to which work avoidance goals were pursued.

### 4.3 Associations of goal profiles with teachers’ self-efficacy beliefs and work-related distress

**Fig 2:**
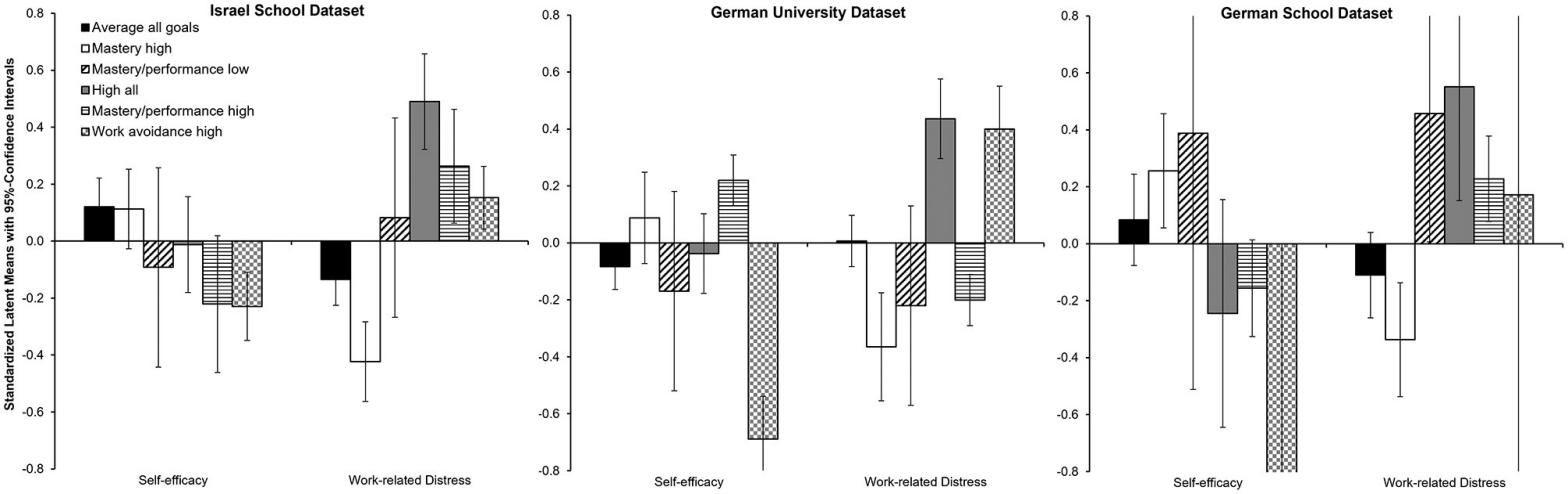
*z*-standardized latent means and 95% confidence intervals for self-efficacy and work-related distress levels in the six profiles per study set.

To determine the explanatory power of the profiles, we conducted equality tests of the means of self-efficacy and work-related distress across the latent profiles. Fig 2 displays standardized latent means and their 95%-confidence intervals, the results of all individual tests are provided in S3 Table in S1 File. The results indicated small differences in self-efficacy between teachers who were classified into different profiles (χ^2^ = 18.3–31.4, *p* < .003). Specifically, teachers in the work avoidance high profile reported lower self-efficacy beliefs than teachers in the average, high all, and mastery high profiles (χ^2^ = 4.3–38.3, *p* < .05). In addition, but only in the German-university study set, participants in the mastery/performance high profile in turn had stronger self-efficacy beliefs than the average and high all profile (χ^2^ = 4.9–16.0, *p* < .05). For work-related distress, we found a medium goal profile membership effect (χ^2^ = 31.1–95.3, *p* < .001). Teachers in the mastery high profile reported least work-related distress compared to teachers from the other profiles (χ^2^ > 4.2, *p* < .05), however, not all differences tests in the German-school study were statistically significant due to the small number of teachers in some profiles. Further, in the German-university study set, teachers in the high mastery profile did not differ statistically significantly from the mastery/performance high profile that was generally characterized by lower levels of work-related distress than the respective profile in the other two study sets. The highest levels of work-related distress were observed in the high all profile.

These differences in self-efficacy beliefs and work-related distress were also evident in structural equation models (see Table 4); profile membership explained 2–9% of the variance in self-efficacy and 7–8% of the variance in work-related distress across the three study sets. Conversely, taking a variable-centered approach by investigating the effect of the individual goals instead of the goal profiles, the individual achievement goals explained 9–25% of the variance in self-efficacy and 12–19% of the variance in work-related distress. Self-efficacy was positively related to mastery approach and performance approach goals and negatively related to performance avoidance and work avoidance goals, while work-related distress was negatively related to mastery goals, and positively to performance avoidance and work avoidance goals. This pattern of results was similar across the three study sets, with two exceptions: work avoidance goals did not reach statistical significance in the Israel-school study set for self-efficacy, and performance avoidance goals were statistically significantly related to increased work-related distress in the German-school study set but not the other two. Comparisons of the confidence intervals showed that the additive main effects of the achievement goals explained substantially more variance in the outcome variables than the goal profiles.

**Table 4:** Results of structural equation models analyzing the relations between goals profiles and individual goals with self-effiacy and work-related distress.

	Israel-school study set (*N* = 1,358)		German-university study set (*N* = 1,765)		German-school study set (*N* = 558)	
	Self-efficacy	Work-related Distress	Self-efficacy	Work-related Distress	Self-efficacy	Work-related Distress
Model 1: Goal profile membership						
Average all goals (♢)	–^a^	–^a^	–^a^	–^a^	–^a^	–^a^
Mastery high (●)	.01 (*p* = .97)	–.11 (*p* < .01)	.06 (*p* = .05)	–.11 (*p* < .01)	.07 (*p* = .18)	–.10 (*p* < .05)
Mastery/performance low (x)	–.03 (*p* = .26)	.04 (*p* = .16)	–.02 (*p* = .48)	–.04 (*p* = .14)	.02 (*p* = .71)	.07 (*p* = .16)
High all (▲)	–.05 (*p* = .19)	.21 (*p* < .01)	.02 (*p* = .57)	.14 (*p* < .01)	–.08 (*p* = .20)	.16 (*p* < .01)
Mastery/performance high (◻)	–.09 (*p* = .02)	.11 (*p* < .01)	.14 (*p* < .01)	–.10 (*p* < .01)	–.09 (*p* = .09)	.15 (*p* < .01)
Work avoidance high (◼)	–.14 (*p* < .01)	.11 (*p* < .01)	–.15 (*p* < .01)	.12 (*p* < .01)	–.25 (*p* < .01)	.02 (*p* = .75)
*R*^2^ with 95% confidence interval	.02 [.01; .04]	.08 [.05; .11]	.05 [.03; .08]	.07 [.04; .09]	.09 [.01; .17]	.07 [.02; .12]
Model 2: Individual achievement goals						
Mastery (learning approach)	.19 (*p* < .01)	–.16 (*p* < .01)	.27 (*p* < .01)	–.14 (*p* < .01)	.29 (*p* < .01)	–.10 (*p* = .20)
Performance approach	.22 (*p* < .01)	–.04 (*p* = .56)	.18 (*p* < .01)	.06 (*p* = .20)	.43 (*p* < .01)	–.13 (*p* = .18)
Performance avoidance	–.22 (*p* = .03)	.01 (*p* = .94)	–.11 (*p* = .02)	–.02 (*p* = .71)	–.53 (*p* < .01)	.32 (*p* < .01)
Work avoidance	–.11 (*p* = .08)	.41 (*p* < .01)	–.13 (*p* < .01)	.27 (*p* < .01)	–.12 (*p* = .04)	.17 (*p* = .01)
*R*^2^ with 95% confidence interval	.09 [.04; .13]	.19 [.13; .24]	.14 [.10; .19]	.12 [.08; .15]	.25 [.14; .36]	.12 [.04; .19]

*Note*. Presented are standardized regression weights. Individual achievement goals, self-efficacy, and work-related distress were estimated as latent variables based on item parcels, χ^2^ ≤ 132.6, CFA ≥ .991, TLI ≥ .985, RMSEA ≤ .051, SRMR ≤ .028.

^a^ The average all profile was used as a reference group.

## 5. Discussion

We analyzed achievement goal profiles of teachers and their meaningfulness by investigating whether psychologically reasonable, coherent, and generalizable goal profiles can be found in different teacher populations, how such profiles are associated with teachers’ self-efficacy and work-related distress, and how powerful such an approach can be compared to a more commonly used variable-centered approach. Strengths of the work include the consideration of multiple large datasets with similar measures from different educational settings, the investigation of goal profiles in a hitherto uninvestigated population, and the explicit comparison of exploratory power between profiles and individual goals.

Using large teacher samples from Israel and Germany as well as schools and universities and data about their mastery, performance approach, performance avoidance, and work avoidance goals, we found psychologically sensible and largely generalizable goal profiles that can be helpful in describing teachers’ achievement goal pursuit. However, most profiles primarily differed by strength of performance goals and had only little explanatory value compared to the individual achievement goals. As such, a more critical picture results from the present study towards using achievement goal profiles in teachers.

### 5.1 Is it possible to identify meaningful achievement goal profiles in teachers?

Regarding our first question, we identified six achievement goal profiles in each of the different study sets. In order to overcome limitations of person-centered approaches, we also investigated the generalizability of the profiles across countries and educational settings. In doing so, we found profiles to be largely comparable across the study sets. While the detected profiles were quite similar across the first two study sets (Israel school teachers, German university teachers), one should note that a less clear pattern emerged for the smallest study set of German school teachers. Only a few teachers were classified into some of the profiles, and goal means in the high all versus work avoidance high profiles differed somewhat more than in the other two study sets. Additionally, selection of the number of profiles was less clear in this sample. It is possible that the smaller sample size might have played some role. There is currently no consensus regarding the minimum required sample sizes for LPAs [for overviews, see 60, 77]. Although the sample sizes of the study sets were above commonly used rules of thumbs of *N* = 500 [59, 60], there is evidence that required sample sizes can vary depending on many aspects such as the number of indicators, the structure of the latent profiles, and covariates [59, 83, 86]. Furthermore, small samples might enable detection of the correct number of profiles [84], but not yield clear patterns for some of the profiles. This generally emphasizes that requirements of LPAs may not always be fulfilled and that one needs to be cautious when running and interpreting such analyses.

The goal profiles that we consistently found showed quantitative as well as qualitative differences. Similar to the meta-analysis of students’ achievement goal profiles by Wormington and Linnenbrink-Garcia [13] and the LPA of vocational training instructors by Kunst et al. [48], we found that the majority of participants were classified into an average all goals profile (39 percent overall) or a high all goals profile (13 percent overall). Further, our analyses documented that the identified profiles mainly differed in mean levels of performance goals but not so much in qualitatively different compositions of the other goals. In particular, the group with strong mastery and strong performance goals and the group with similarly strong mastery goals but much less strongly pursued performance goals were very similar to the respective profiles reported in Wormington and Linnenbrink-Garcia [13], and line up with initial theorizing into the nature of achievement goal pursuit from a multiple goal perspective (pursuit of mastery *and* performance goals; e.g., [12]). Finding such profiles in different populations strongly speaks to their existence and to their sensibility in describing differences in achievement goal pursuit.

However, comparing our findings to these previous works, it also needs to be noted that the profiles that we found mostly did not strongly differ regarding mastery goals. In fact, nearly all participants were in a profile with similarly strong mastery goals, with no profile with stronger mastery goals standing out having been identified. This finding may be well in line with the generally strong mastery goals reported for the teacher population and could indicate differences in goal pursuit between student and teacher populations [with teachers strongly valuing learning already; see also 10, 29]. Investigation of our sample characteristics also showed indications of such a general ceiling effect of mastery goals (reflected in large mean values and small standard deviations, see Table 2). Furthermore, while Wormington and Linnenbrink-Garcia [13] conflated performance avoidance and work avoidance goals, we investigated them separately from each other. This proved to be very helpful in the present work, as we found substantial differences in goal profiles based on teachers’ work avoidance goals (particularly regarding the high all vs. the mastery/performance high profiles). From a psychological perspective, it makes sense that teachers can pursue strong mastery and performance and either strong and little work avoidance goals. Our findings also point to the distinctiveness of work avoidance goals from other goals (particularly performance avoidance goals; see [85]) as well as the merits of including these goals for describing the pursuit of the different self-related aims that teachers may hold in achievement situations at work. Finally, as in Wormington and Linnenbrink-Garcia [13], we did not find goal profiles that differed between performance approach and performance avoidance goals, which is in line with the strong correlation typically reported for this type of goal, particularly in research on teachers [see 28, 32]. While it stands to reason that different groups of people should be able to be identified that differ in the extent to which they pursue performance approach and avoidance goals, these differences are likely much smaller than the differences regarding mastery and work avoidance goals, which is why they may not be readily found in latent profile analyses when also including these goals. Taken together, this suggests that combining performance approach and performance avoidance goals in profile analyses might not be particularly useful—at least when other achievement goals (such as mastery or work avoidance goals) are also considered.

In sum, goal profiles can theoretically be considered as sensible to describe how individuals actually pursue goals (multiple goals at the same time and to different strengths), however, the large proportion of profile differences due to mean level differences in performance goals might question the usefulness of the LPA approach (opposed to merely describing the strength to which they pursued the different achievement goals) for describing differences between different groups of people in the quality of their motivations.

### 5.2 How useful are teachers’ achievement goal profiles in explaining differences in teachers’ self-efficacy beliefs and work-related distress compared to individual goals?

Regarding our second and third research questions, we did find differences in self-efficacy beliefs and work-related distress in teachers based on the goal profiles they were assigned to. However, these differences were primarily found for those profiles such as mastery high or work avoidance high in which one type of goal was substantially stronger than the others. Moreover, results for these profiles were in line with the positive associations typically reported under a variable-centered view for mastery goals, as well as with the negative associations for work-avoidance goals, with favorable levels of self-efficacy beliefs and work-related distress. It should also be noted that while the general pattern of results regarding the differences in self-efficacy and work-related distress were largely similar across the three study sets, this was not the case for the mastery/performance high profile. Participants that were classified into this profile in the German-university sample exhibited higher levels of self-efficacy and lower levels of work-related distress than participants classified into the same profile in the other two study sets. This might be a function of the mastery/performance high profile that was not characterized by the same level of mastery goal across the three study sets (but primarily so within the German-university sample). As such, for the purpose of explaining differences in self-efficacy and work-related distress, knowledge of a teacher being classified into this profile proved not to be particularly helpful without further information on which study set the profile belonged to—or, more specifically, the exact levels of the mastery goals.

This finding was also evident in general, when comparing the profile approach to the main effects of the individual goals. Importantly, we found here that the inter-individual differences between teachers in their self-efficacy and work-related distress could be primarily traced back to individual goals and their additive effects. Compared to these effects, the goal profiles yielded hardly any explanatory value for teachers’ self-efficacy and work-related distress. Such direct comparisons between person-centered and variable-centered analyses are rare and an important source of information for evaluating the merits of both approaches to the study of achievement goals [43, 44, 87]. However, it should be noted that the amount of explained variance may not be a fair comparison criterion for the two methods, as goal profile membership contains restricted variance compared to the strength to which the individual goals are pursued. Nevertheless, as researchers typically use these approaches with the ultimate aim of explaining differences in outcome variables, the amount of explained variance is a highly relevant criterion with regard to application purposes. Furthermore, the small amounts of explained variances raises concerns about using them as a basis for profile-specific interventions. Taken together, our findings on the comparison of these two approaches imply that asking “which profile is more beneficial” may not be a very beneficial question—especially when this question is asked with a pure exploratory focus and the analysis is not guided by justified hypotheses on the effects of these profiles (as is frequently the case in general applications of person-centered approaches). Instead, the individual achievement goals seem to be more useful in explaining differences in teachers’ cognitions and experiences.

Regarding the multiple-goal perspective, this means that such an approach is likely adequate for describing actual goal pursuit, the core premise that functionality and effects of achievement goals would change substantially depending on their composition [see 12, 33] cannot be confirmed with the present work. Instead, the main effects that we found are rather in favor of a mastery-goal perspective—similar to the most favorable effects having been reported for the high-mastery goal profiles by Wormington and Linnenbrink-Garcia [13]. While we only investigated self-efficacy beliefs and work-related distress in the teacher population, this finding may also hold true for other variables and populations, and thus warrants a more skeptical view on the merits of achievement goal profiles in explaining cognitions and experiences of individuals compared to the main effects of the individual goals.

### 5.3 Limitations and future research

Interpreting these findings, there are limitations of the current work that need to be borne in mind. First, while we used data from different studies and different institutions, not all of the measures were identical (albeit very similar). As such, it is likely that with the measures tapping on slightly different aspects, we could not have found perfectly identical profiles across the different study sets in the first place. This means that our findings on the generalizability of the different goal profiles across the different study sets can be expected to underestimate the actual similarities.

Second, we only investigated teachers from Israel and Germany. While the inclusion of different countries and types of institutions is a great strength of the present investigation, future research could profit from including even more different (e.g., Eastern) countries or other Western countries that place greater emphasis on teacher evaluation through high-stakes testing (e.g., the US). This could offer a very interesting perspective regarding the generalizability of different goal profiles.

Third, our findings are limited in that we only included mastery, performance approach, performance avoidance, and work avoidance goals. While this lines up well with the majority of past research on teachers’ goals, roots in the fundamental work by Butler [22], and allows sensible comparisons to investigations into goal profiles in students, it should be noted that the profiles found might strongly depend on the different goals used (as indicated by our results for work avoidance goals). Future research might therefore also consider other goals, such as relational goals that have proven powerful in describing the motivations for teaching [see 88] and have also been used successfully in first investigations into academic and social goal profiles in students [see 87, 89].

Fourth, we used LPAs as a person-centered approach to studying teacher motivation. It should be considered that the background of person-centered analyses is to focus on the individual rather than on the population [thus the term “person-centered”; e.g., 13, 90]. However, in most studies, the “person-centered” approach is applied to compare subgroups of individuals on outcome variables [this is particularly true for research on goal profiles; e.g., 15, 17]. In this case, the term “person-centered approach” is strictly speaking misleading and it might be more appropriate to speak of “pattern-centered or categorical latent variable” analyses that allow for the identification and comparison of subgroups within a given population. In contrast, it may be argued that person-centered analyses in a strict sense require variance within individuals and as such, multiple measurement occasions [91] to investigate the interplay of variables within persons or their shift between identified subgroups. Even though this terminology might therefore be considered as somewhat inaccurate, we used the term “person-centered approach” in the present work given that it is commonly used in the achievement goal literature when referring to analyses into goal profiles.

### 5.4 Conclusions

Taken together, our findings indicate that results from latent profile analyses may be fuzzier than often thought. Our findings imply that goal profiles make sense and can be a good approach to describing teachers’ actual goal pursuit, with psychologically meaningful profiles that we consistently found across different study sets (albeit heavily relying on differences in the strength of performance goals instead of further qualitative differences in motivations). Nevertheless, finding goal profiles may not always be easy and depend on relevant methodological features such as the sample size. Moreover, the usefulness of this approach for explaining differences in teachers’ cognitions and experiences may be limited as goal profiles had little explanatory value compared to individual goals. As such, we consider goal profile analyses valuable for describing goal pursuit, but we also see danger in the unreflective use of such analyses and are skeptical about their added value for research on the correlates of teachers’ achievement motivation and achievement goal research in general.

## Supporting information

S1 File (PDF)
