# Supplementary material for: Merits and limitations of latent profile approaches to teachers’ achievement goals: A multi-study analysis
Source: PLoS One. 2023 Apr 20;18(4):e0284608. doi: 10.1371/journal.pone.0284608 (PMC10118172; doi:10.1371/journal.pone.0284608)
Supplement: S1 File — (PDF) [file pone.0284608.s001.pdf]

Martin Daumiller, Stefan Janke, Ruth Butler, Oliver Dickhäuser, & Markus Dresel

Merits and Limitations of Latent Profile Approaches to Teachers' Achievement Goals: A Multi-Study Analysis

2023

PLOS ONE

https://doi.org/10.1371/journal.pone.0284608

Supplementary Materials

This supplement contains the following:

* Table S1

* Table S2

* Table S3

* Figure S1

* Figure S2

* Figure S3

Table S1

Overview of LPA Profile Solutions on the Level of the Overall Dataset

Number of profiles	Fit indices			Likelihood ratio tests (*p* values)			Number of teachers in profiles								
	BIC	SSBIC	AIC	VLMR	LMR	Entropy	*1*	*2*	*3*	*4*	*5*	*6*	*7*	*8*	*9*
Overall dataset															
2	38,902	38,861	38,821	<.001	<.001	.71	2,376	1,305							
3	38,527	38,470	38,415	<.001	<.001	.77	2,372	1,058	251						
4	38,222	38,149	38,079	<.001	<.001	.70	1,611	1,340	580	150					
5	38,086	37,997	37,912	.004	.004	.66	1,557	1,017	555	408	144				
6	37,937	37,832	37,732	.005	.004	.69	1,441	926	552	485	211	66			
7	37,880	37,759	37,644	.40	.41	.71	1,451	883	546	502	197	79	23		
8	37,825	37,689	37,558	.21	.21	.70	1,323	940	491	366	210	180	141	30	
9	37,777	37,624	37,479	.32	.32	.70	1,224	850	491	348	304	250	153	37	24

Note. BIC = Bayesian information criterion. SSA-BIC = sample-size-adjusted BIC. AIC = Akaike information criterion. VLMR = Vuong-Lo-Mendell-Rubin likelihood ratio test. LMR = Lo-Mendell-Rubin adjusted likelihood ratio test.

Table S2

Results of Structural Equation Models Analyzing the Relations Between Goals Profiles and Individual Goals with Self-effiacy and Work-related Distress

	Model 1: Goal profiles		Model 2: Individual goals	
	Self-efficacy	Work-related Distress	Self-efficacy	Work-related Distress
Goal profile membership				
Average all goals (◊)	–[a]	–[a]		
Mastery high (●)	.07 ($p = .001$)	–.11 ($p < .001$)		
Mastery/performance low (x)	–.02 ($p = .41$)	.01 ($p = .66$)		
High all (▲)	–.01 ($p = .74$)	.17 ($p < .001$)		
Mastery/performance high (□)	.11 ($p < .001$)	–.02 ($p = .25$)		
Work avoidance high (■)	–.10 ($p < .001$)	.12 ($p < .001$)		
Individual achievement goals				
Mastery (learning approach)			.25 ($p < .001$)	–.14 ($p < .001$)
Performance approach			.23 ($p < .001$)	–.01 ($p = .837$)
Performance avoidance			–.20 ($p < .001$)	.07 ($p = .03$)
Work avoidance			–.14 ($p < .001$)	.29 ($p < .001$)
R^2 with 95% confidence interval	.028 [.016; .040]	.059 [.041; .077]	.135 [.104; .166]	.129 [.102; .157]

Note. $N = 3{,}681$ teachers. Presented are standardized regression weights. Individual achievement goals, self-efficacy, and work-related distress were estimated as latent variables based on item parcels, $\chi^2 \leq 116.49$, CFA ≥ .998, TLI ≥ .996, RMSEA ≤ .016, SRMR ≤ .007.

[a] The average all profile was used as a reference group.

Table S3

Results of BCH Tests Regarding Differences in Self-Efficacy ad Work-related Distress Between the Identified Profiles

	[1]	[2]	[3]	[4]	[5]	[6]
Israel-school study set						
[1] Average all goals (◊)		**4.22**	3.82	**38.54**	**8.52**	**15.96**
[2] Mastery high (●)	0.00		**7.85**	**68.29**	**16.92**	**31.11**
[3] Mastery/performance low (x)	0.57	0.47		2.97	0.13	0.03
[4] High all (▲)	1.21	1.23	0.00		2.08	**8.37**
[5] Mastery/performance high (□)	3.13	2.99	0.60	0.65		0.07
[6] Work avoidance high (■)	**12.34**	**10.33**	1.69	**4.35**	0.15	
German-university study set						
[1] Average all goals (◊)		**14.16**	**2.26**	**13.94**	**10.20**	**10.25**
[2] Mastery high (●)	3.57		0.15	**45.07**	1.88	**35.09**
[3] Mastery/performance low (x)	0.07	1.02		**10.36**	0.04	**9.62**
[4] High all (▲)	1.33	0.55	0.49		**39.81**	0.09
[5] Mastery/performance high (□)	**16.04**	2.20	2.51	**4.92**		**32.86**
[6] Work avoidance high (■)	**23.43**	**38.33**	3.20	**26.68**	**75.27**	
German-school study set						
[1] Average all goals (◊)		**13.06**	0.04	2.25	**8.10**	0.24
[2] Mastery high (●)	2.80		**3.87**	**19.11**	2.72	0.30
[3] Mastery/performance low (x)	0.69	0.00		0.56	2.24	0.13
[4] High all (▲)	0.24	**7.53**	0.41		6.47	0.00
[5] Mastery/performance high (□)	1.51	1.07	0.06	2.40		0.09
[6] Work avoidance high (■)	**14.37**	**22.84**	14.68	**18.33**	21.05	

Note. $N = 1{,}358$, $N = 1{,}765$, $N = 558$, respectively. Presented are the results of the equality tests of means in self-efficacy (lower triangular matrices) and work-related distress (upper triangular matrices) across the identified profiles per study set using the BCH procedure. Statistically significant parameters ($p < .05$) are boldfaced.

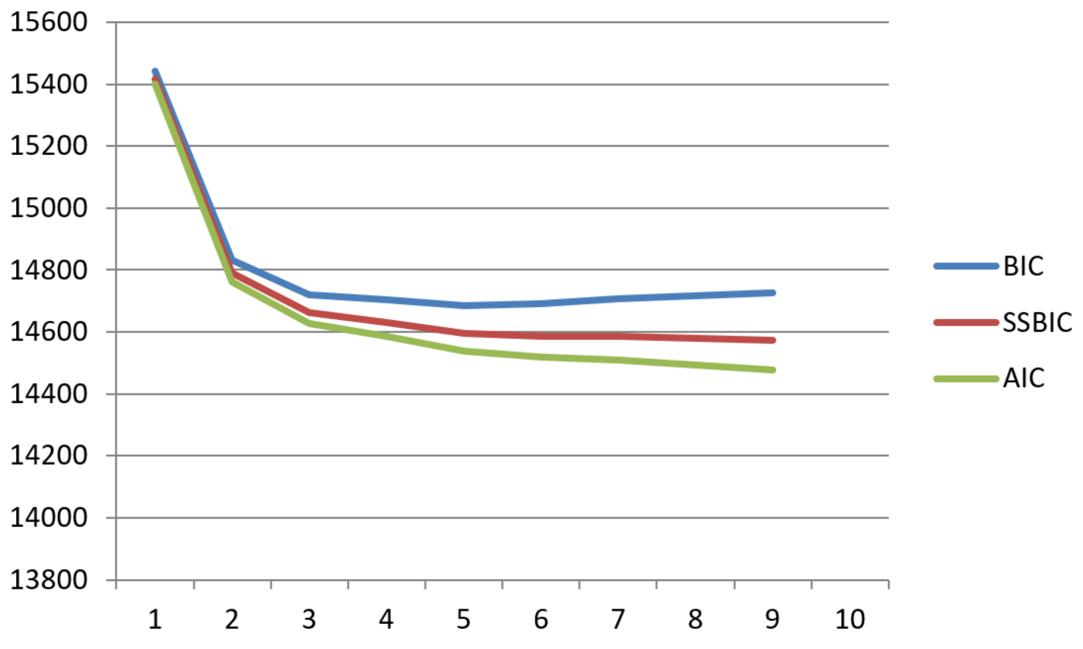
15600
15400
15200
15000
14800
14600
14400
14200
14000
13800
1
2
3
4
5
6
7
8
9
10
BIC
SSBIC
AIC

(a)

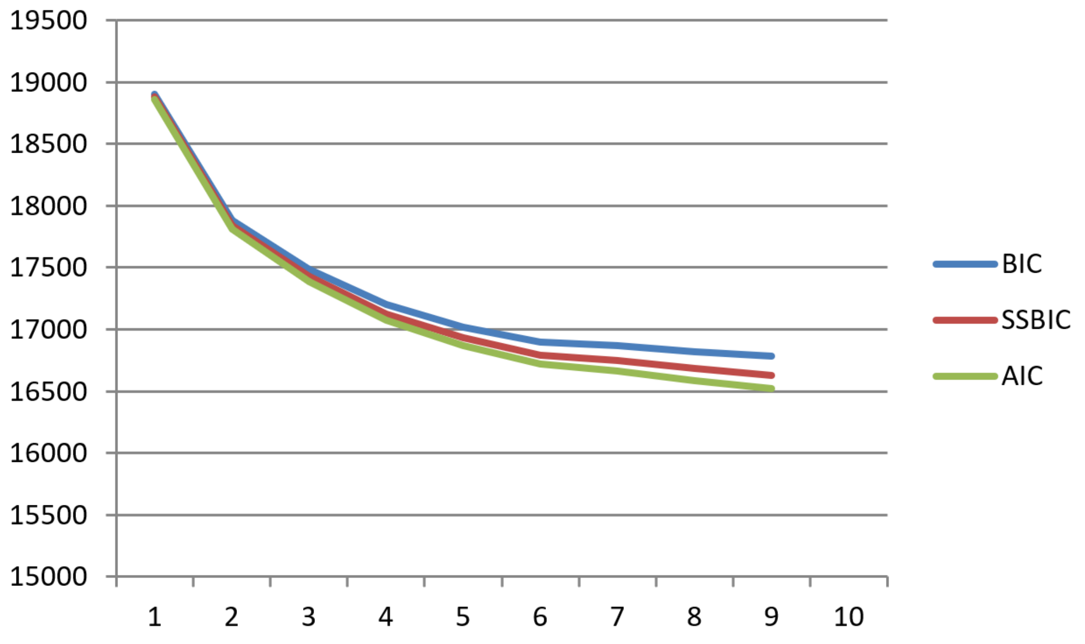
19500
19000
18500
18000
17500
17000
16500
16000
15500
15000
1
2
3
4
5
6
7
8
9
10
BIC
SSBIC
AIC

(b)

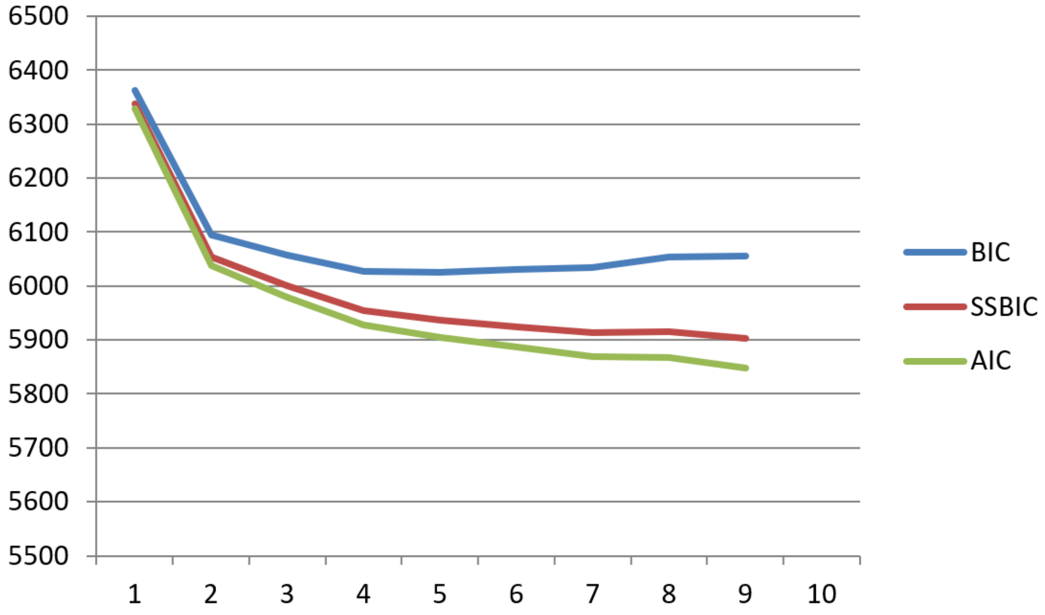

(c)

Figure S1. Visualization of the BIC, SSBIC, and AIC fit indices for the different profile solutions across the (a) Israel-school study set, (b) German-university study set, and (c) German-school study set.

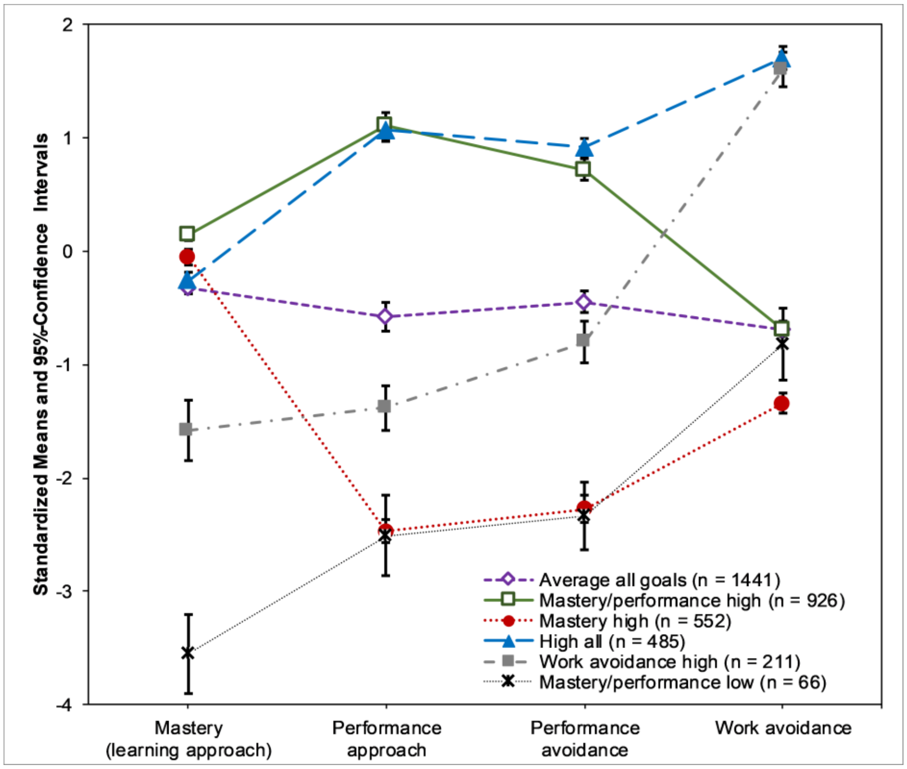

Figure S2. Goal means for the six latent profiles in the joint overall dataset (presented are standardized means with 95% confidence intervals)

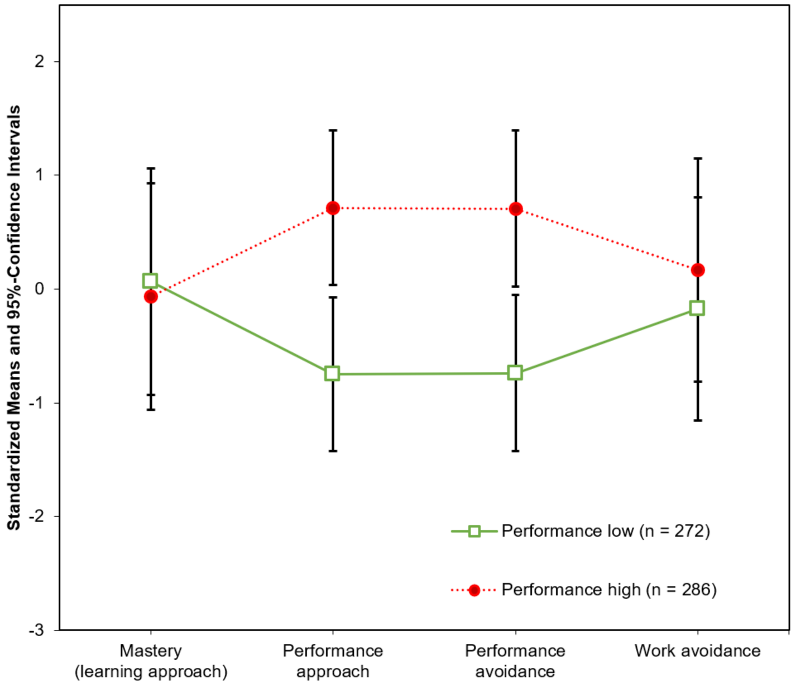

(a)

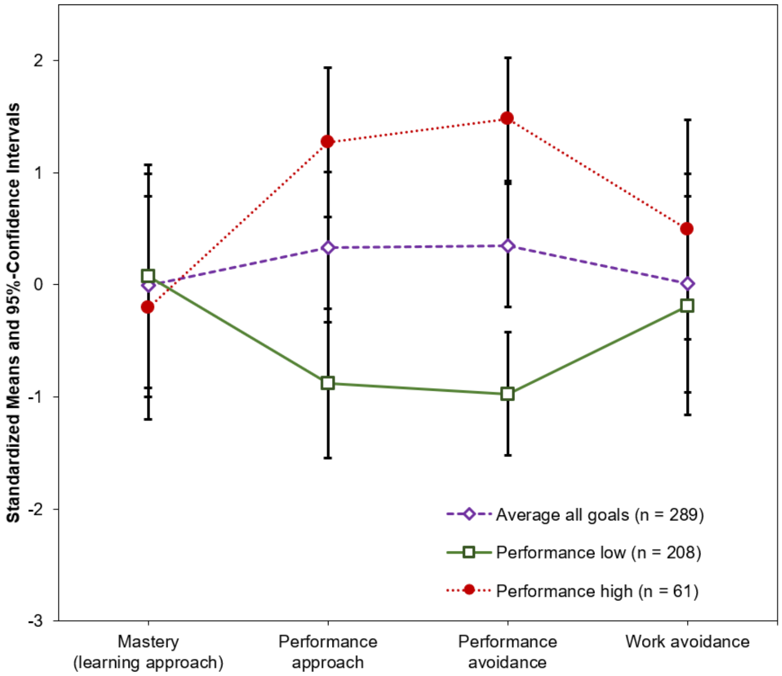

(b)

Figure S3. Goal means of the supplemental (a) two and (b) three profile solutions with the German-school study set (presented are standardized means with 95% confidence intervals)
